# Trends in the incidence of recurrent stroke at 5 years after the first-ever stroke in rural China: a population-based stroke surveillance from 1992 to 2017

**DOI:** 10.18632/aging.101862

**Published:** 2019-03-19

**Authors:** Wenjuan Zhao, Jialing Wu, Jie Liu, Yanan Wu, Jingxian Ni, Hongfei Gu, Jun Tu, Jinghua Wang, Zhongping An, Xianjia Ning

**Affiliations:** 1 Department of Neurology, Tianjin Huanhu Hospital, Tianjin 300350, China; 2 Tianjin Key Laboratory of Cerebral Vascular and Neurodegenerative Disease, Tianjin 300350, China; 3 Department of Neurology, Tianjin Medical University General Hospital, Tianjin 300052, China; 4 Laboratory of Epidemiology, Tianjin Neurological Institute, Tianjin 300052, China; 5 Tianjin Neurological Institute, Key Laboratory of Post-Neuroinjury Neuro-repair and Regeneration in Central Nervous System, Ministry of Education and Tianjin City, Tianjin 300052, China; 6 Department of Neurology, Tianjin Haibin People’s Hospital, Tianjin 300280, China

**Keywords:** stroke, recurrence, epidemiology, trend

## Abstract

Recent data on the incidence and trends for recurrent strokes in China are scarce. We assessed the temporal trends in recurrent stroke incidence using in rural China. The age-standardized incidences of recurrent stroke, within 5 years of the incident stroke event, were estimated for 3 time periods: 1992–1998, 1999–2005, and 2006–2012. Among the 768 documented incident stroke cases, 26.3% of the patients experienced recurrent stroke within 5 years. The overall age-adjusted recurrent stroke incidence was 43.93 per 100,000 person-years (1992–2012). During the 2006–2012 period, the recurrent stroke incidence per 100,000 person-years was 107.79 in men, and 557.76 in individuals ≥65 years old. There were significant upward tendencies observed in this population across sex, age, or type of stroke (except for among individuals ≥65 years old with incident intracerebral hemorrhages). Compared with the recurrent stroke incidence observed in the 1992–1998 period, that observed during the 2006–2012 period was more than 3-fold higher; the greatest increase (6.8-fold) was observed in women. These findings suggest an urgent need to improve risk factor management and implement appropriate medical resources to contain this upward trend in recurrent stroke incidence and reduce the overall stroke burden in China.

## Introduction

Recurrent strokes are more likely to be disabling or fatal than incident strokes, and population-based studies have documented that recurrent strokes account for more than 30% of all strokes [1]. Therefore, the prevention of recurrent strokes is important to both patients and society. Strokes are one of the leading causes of death and disability, worldwide [2], accounting for 9% (almost 4.4 million) of all deaths each year [3]. Over the past 2 decades, there has been a notable increase (84%) in the absolute number of stroke survivors globally, despite a decreasing incidence of strokes in high-income countries [4]. However, the number of stroke survivors <75 years old is approximately 30% higher in low- and middle-income countries than in high-income countries [4].

In most Western countries, stroke-related mortality has significantly declined over the past few decades, and the number of stroke survivors has been increasing [5–7]. Thus, the risk of recurrent strokes is becoming more important due to their increased likelihood of causing disability and death [8–11]. In contrast with global trends, the incidence of first-ever strokes has dramatically increased in China [12,13], especially in rural China [14,15]. China, a developing country with one-fifth of the world’s population, reports 2.5 million incident stroke cases each year and has approximately 7.5 million stroke survivors. Further, the rate of recurrent stroke also remains high at 11.2% [11].

However, data regarding trends in the rate of stroke recurrence, especially in conjunction with the increased numbers of incident stroke cases, remains unknown in China. This is compounded by the lack of the population-based studies addressing the incidence of recurrent strokes. Thus, we explored trends in the incidence of recurrent strokes within 5 years of the incident event, in a rural population in China.

## RESULTS

### Demographic features of stroke patients (1992–2012)

A total of 768 incident strokes were reported, including 461 (60%) in men and 307 (40%) in women. Of these, 202 (26.3%) patients experienced recurrent stroke within 5 years, including 28.0% of the male and 23.8% of the female patients. Patients suffering recurrent strokes were younger (mean, 64.92 years) and had less education (mean, 3.06 years) at the time of the incident stroke than patients not suffering recurrent strokes. Patients ≥65 years old accounted for 51.4% of the recurrent stroke victims, and 88.3% of the victims did not achieve middle school education. Recurrent strokes were reported in 32% of patients experiencing incident ICH and in 25.2% of patients with incident IS (Table 1).

**Table 1:** The descriptive characteristics of patients with first-ever stroke by gender and period in the Tianjin Brain Study.

Demographical Characteristics	Total	Recurrence within 5 years	Non-recurrence	P
Gender, n (%):				0.195
Men	461 (60.0)	129 (28.0)	332 (72.0)	
Women	307 (40.0)	73 (23.8)	234 (76.2)	
Total	768 (100)	202 (26.3)	566 (73.7)	
Age of stroke, means (SD), years	64.92 (11.82)	66.25 (11.08)	64.45 (12.05)	
Age groups, n (%)				0.164
< 45 years	42 (5.5)	6 (3.0)	36 (6.4)	
45~64 years	331 (43.1)	87 (43.0)	244 (43.1)	
≥ 65 years	395 (51.4)	109 (54.0)	286 (50.5)	
Education attainment, means (SD), years	3.06 (3.21)	2.98 (2.81)	3.10 (3.35)	
Education groups, n (%):				0.743
0 year	313 (40.8)	71 (22.7)	242 (77.3)	
1 to 6 years	365 (47.5)	116 (31.8)	249 (68.2)	
≥7 years	90 (11.7)	15 (16.7)	75 (83.3)	
Time of following-up, means (SD), years	3.61 (1.69)	2.14 (1.37)	4.13 (1.48)	
Types of first-ever stroke, n (%)				0.114
ICH	125 (100)	40 (32.0)	85 (68.0)	
IS	643 (100)	162 (25.2)	481 (74.8)	
Diagnosis by neuroimaging, n (%):	191 (61.4)	120 (59.4)	71 (65.1)	

SD indicated standard deviation; ICH=Intracerebral hemorrhage; IS=Ischemic stroke.

### Age-adjusted incidence of recurrent stroke (1992–2012)

Overall, the age-adjusted incidence of recurrent stroke was 43.93/100,000 person-years (95% CI, 36.66–51.20); 57.85 (95% CI, 39.47–76.23) for men and 30.92 (95% CI, 22.10–39.74) for women. For patients <65yearsold, the corresponding incidence was 19.64; for those ≥65years old, it was 314.61. The highest incidence (557.76) was during the 2006–2012 period for individuals ≥65 years old. There was a significant upward tendency in the observed incidences during the 3 study stages (P<0.001), with similar findings for men and women (P < 0.001), for those <65 or ≥65 years old, for those experiencing incident ICH (P = 0.033), and for those experiencing incident IS (P < 0.001) (Table 2).

**Table 2:** Age-adjusted incidence per 100000 person-years of the recurrent stroke during 1992 to 2012 by demographical feature (95% CI).

Category	1992-2012	1992-1998	1999-2005	2006-2012	P for trend
Gender:					
Men	57.85 (39.47, 76.23)	18.93 (7.29, 30.57)	43.42 (25.53, 61.31)	102.79 (77.13, 128.45)	<0.001
Women	30.92 (22.10, 39.74)	8.19 (0.27, 16.11)	18.04 (6.06, 30.02)	62.06 (41.15, 82.97)	<0.001
Total	43.93 (36.66, 51.20)	13.61 (6.51, 20.71)	30.41 (19.60, 41.19)	81.23 (64.73, 97.73)	<0.001
Age group:					
< 65 years	19.64 (14.49, 24.79)	5.54 (0.76, 10.32)	12.25 (5.41, 19.95)	38.46 (26.41, 44.67)	<0.001
≥65 years	314.61 (255.52, 373.70)	103.58 (42.43, 164.73)	232.74 (143.38, 322.10)	557.76 (428.28, 687.24)	<0.001
Types of first-ever stroke:					
ICH	8.68 (5.45, 11.91)	4.25 (0.29, 8.21)	5.82 (1.33, 10.31)	15.37 (8.18, 22.56)	0.033
IS	35.25 (28.72, 41.78)	9.37 (3.49, 15.25)	25.13 (15.33, 34.93)	65.86 (50.98, 80.74)	<0.001

ICH=Intracerebral hemorrhage, IS=Ischemic stroke.

### Relative risk of age-adjusted incidences of recurrent stroke

Table 3 shows that the age-adjusted incidences of the recurrent stroke increased 1.36-fold during the 1999–2005 period (P=0.006) and 5.03-fold during the 2006–2012 period (P<0.001) compared with the 1992–1998 period. A 1.35-fold increase in the age-adjusted incidence of recurrent stroke was also observed during the 2006–2012 period relative to the 1999–2005 period (P<0.001). The trend in men was similar to the overall trend; however, in women, the greatest increase in the age-adjusted incidence of recurrent stroke was 6.82-fold during the 2006–2012 period compared with the 1992–1998 period (P<0.001).

**Table 3:** Relative risk of the recurrent stroke during 1992 to 2012 by demographical feature.

Category	1992-1998	1999-2005 vs 1992-1998	2006-2012 vs 1992-1998	2006-2012 vs 1999-2005
Relative Risk (95% CI):				
Gender:				
Men	1.00	2.37 (1.13, 4.98)*	5.56 (2.85, 10.85)*	2.55 (1.71, 3.81)*
Women	1.00	2.34 (0.72, 7.58)	7.82 (2.78, 22.04)*	3.35 (1.61, 6.98)*
Total	1.00	2.36 (1.26, 4.43)*	6.03 (3.44, 10.58)*	2.35 (1.45, 3.78)*
Age group:				
< 65 years	1.00	2.30 (0.80, 6.62)	5.32 (2.06, 13.73)*	2.31 (1.16, 4.63)*
≥65 years	1.00	2.25 (1.11, 4.56)*	5.42 (2.87, 10.24)*	2.41 (1.54, 3.78)*
Types of first-ever stroke:				
ICH	1.00	1.55 (0.44, 5.50)	4.08 (1.38, 12.07)*	2.63 (1.05, 6.63)*
IS	1.00	2.59 (1.24, 5.38)*	6.81 (3.52, 13.17)*	2.63 (1.67, 4.14)*

* indicated P<0.05

### Age-adjusted incidence of recurrent stroke by age group

There was an upward trend in the age-adjusted incidence of recurrent stroke during the 3 study stages, regardless of patient sex or incident stroke type in patients <65 or ≥65 years old, except for incident ICH in patients ≥65 years old (Table 4).

**Table 4:** Age-adjusted incidence per 100000 person-years of the recurrent stroke during 1992 to 2012 by demographical feature and age (95% CI).

Category	1992-1998	1999-2005	2006-2012	P for trend
< 65 years:				
Gender:				
Men	5.53 (0, 12.15)	17.52 (5.51, 29.53)	49.16 (30.38, 67.94)	<0.001
Women	5.56 (0, 12.48)	6.75 (0, 14.55)	28.23 (13.22, 43.24)	0.001
Subtypes of first-ever stroke:				
ICH	1.28 (0, 3.57)	3.43 (0, 7.27)	9.85 (3.75, 15.95)	0.006
IS	4.26 (0.07, 8.45)	8.82 (2.65, 14.99)	28.61 (18.20, 39.02)	<0.001
≥65 years:				
Gender:				
Men	168.33 (57.49, 279.17)	332.06 (178.45, 485.67)	433.96 (271.91, 596.01)	0.015
Women	37.48 (0, 89.24)	143.80 (45.58, 242.02)	439.01 (276.92, 601.10)	<0.001
Types of first-ever stroke:				
ICH	37.34 (0.61, 74.07)	25.90 (16.43, 35.37)	76.78 (28.62, 124.94)	0.141
IS	66.24 (17.32, 115.17)	206.84 (180.13, 233.55)	480.99 (360.70, 601.28)	<0.001

ICH=Intracerebral hemorrhage, IS=Ischemic stroke.

## DISCUSSION

To our knowledge, this is the first report describing the temporal trends in recurrent stroke incidence in China based on a prospective, population-based study. In this study, we assessed the age-standardized recurrent stroke incidence within 5 years of the incident stroke in a rural population in China. Of those experiencing a documented incident stroke within the study period, 26.3% experienced a recurrent stroke within 5 years. This yielded an overall age-adjusted recurrent stroke incidence of 43.93/100,000 person-years. During the 2006–2012 period, the incidence was as high as 107.79/100,000 person-years in men and 557.76/100,000 person-years in individuals ≥65 years old. The recurrent stroke incidence demonstrated a significant upward trend during the 3study periods, regardless of sex, age, or incident stroke subtype (except for recurrent strokes among individuals ≥65 years old who experienced ICH as their incident stroke event). Compared with the recurrent stroke incidence during the 1992–1998 period, the incidence during the 2006–2012 period was >3-fold higher, with the greatest increase (6.8-fold) observed in women.

Recurrent strokes cause significantly higher rates of mortality and disability than the incident events [9–11,16]. Thus, the increasing incidence of first-ever strokes and the decreasing mortality rate associated with incident events further augments the hazards associated with recurrent strokes [9,17,18]. Patients surviving incident stroke events also have significantly higher risks of recurrent strokes than the general population [19]. However, studies show considerable variation in the short- and long-term risks of recurrent strokes [20]. For example, the 5-year cumulative risk of stroke recurrence has been variously reported in population-based studies as19% (Manhattan, USA), 29% (Rochester, USA), 30% (Oxfordshire, UK), and 32% (Perth, Australia) [21–24].

In the present study, we assessed the person-year incidence of recurrent stroke in a rural, low-income population, and estimated it to be 43.93/100,000 person-years over a 21-year period. However, there was a much higher incidence among stroke patients aged ≥65 years (314.61/100,000 person-years) during the same period. Further, during the 2006–2012 period, the incidence was 107.79/100,000 person-years in men and 557.76/100,000 person-years in individuals ≥65 years old. This high incidence of recurrent strokes occurred during a period with a high incidence of first-ever strokes in the study population [25,26].

Stroke is a cerebrovascular disorder caused primarily by atherosclerotic factors, other pathogenic factors accounted for a few parts, such as Anderson-Fabry disease (4%), etc [27,28]. Poor management of conventional risk factors, including hypertension, diabetes, overweight, current smoking, and alcohol consumption, may be considered the cause of the rapid increases in the occurrence of both incident and recurrent strokes in rural China. Lower rates of awareness, treatment, and control for hypertension in this population (which were 61.1%, 43.8%, and 12.0% in 2011, respectively) can partly explain the great increasement of the incidence of recurrent strokes in this low-income population [29]. Moreover, the previous studies indicated that patients with baseline SBP ≥120 and ≤180 mmHg and lower total cholesterol level, and those who did not receive cardiovascular drugs had a worse outcome [30]. Vascular inflammation related to hypertension attributed a pathogenetic role to regulate and activate of inflammation-related cells and various inflammatory mediators, the reduction of low-grade inflammation in hypertension may be an important target in order to reduce the cardiovascular morbidity and mortality [31].

A few previous studies also demonstrated trends in the incidence of recurrent stroke. The 4-year cumulative risk of recurrent IS in Sweden was 11.8% in men and 9.8% in women during a 5-year period (2002–2006) [10]; the values from previous studies were slightly lower [9,17,18]. According to another Swedish study, the risk of recurrent IS decreased by 55%over time in younger stroke patients [32]. The Swedish Stroke Register (Riks stroke) reported a downward trend in the 1-year risk of recurrent IS between the 1998–2001 and 2007–2010 time periods, with a RR reduction of 20.0% [32]. Similar trends have been reported in Taiwan and Italy [33,34]. In a population-based study, a 36% decline in stroke recurrence was observed in 6,700 stroke patients between the 1995–1998 and 2004–2008 periods [35]. Further, stroke patients hospitalized in 2001 in Scotland had a 27% lower risk of recurrence than those hospitalized due to strokes in 1986 [36].

Unlike the aforementioned studies, the current study indicated a significant upward trend in the incidence of recurrent strokes during the 3 study stages, regardless of patient sex, age, or incident stroke subtype. Compared with the recurrent stroke incidence for the 1992–1998 period, the incidence had increased >3-fold during the 2006–2012 period, including a 6.8-fold increase in women. Increases in the incidence of first-ever strokes in the study population were consistent with the rise in the incidence of recurrent strokes. Additionally, poor management of the associated risk factors in this low-income population [25,26] and a lower frequency of statin or antiplatelet drug use after IS likely contributed to the increase in the recurrent stroke risk [37].

This study has 2 main strengths. First, the study involved a rigorous, population-based, prospective design that included strict case ascertainment, in-person neurologist verification, and high follow-up rates. Second, we evaluated the recurrent stroke incidence using person-years. However, the study also had several limitations. First, the limited sample size was not representative of the general population of China. Thus, these findings are not suitable for other population. The second limitation was the low frequency of stroke diagnoses using neuroimaging in this patient population, which may affect the classification of stroke subtypes. However, all patients included in this study were symptomatic stroke; patients with silent strokes were excluded. Thus, the incidence of recurrent strokes may have been underestimated actually. Moreover, we also assessed recurrent stroke incidence trends based on overall rates of recurrence rather than on the rates of stroke subtypes to minimize the impact of this limitation.

## CONCLUSIONS

This report describes the temporal trends in the incidence of recurrent strokes in a rural, low-income population in China. Among individuals suffering from an incident stroke, 26.3% experienced recurrent strokes within 5 years. Further, the incidence of recurrent strokes increased in each of the 6-year study stages, regardless of patient age, sex, or incident stroke subtype (except within the oldest age group who experienced incident ICH). The high incidence of first-ever strokes and poor risk factor management in the study population may explain the upward trend in recurrent stroke incidence. These findings suggest an urgent need to improve risk factor management and implement appropriate medical resources to control the upward trend in recurrent stroke incidence and the overall burden of strokes in China’s low-income population.

## MATERIALS AND METHODS

### Study population

The study population and design were previously described [14,15,25]. Briefly, the Tianjin Brain Study was a population-based stroke surveillance study that began in 1985 and was conducted in Tianjin, China. The study included the 15,438 residents of the township. The majority (95%) of the adults in the township were low-income farmers with an annual per capita income of < 100 USD (1990) to < 2000 USD (2015) [26]. In 1991, the illiteracy rates for residents 35–74 years old were 30% for men and 40% for women. The population characteristics remained stable over the study period [29].

The ethics committee of Tianjin Medical University General Hospital (TMUGH) approved the study protocol and each participant provided written informed consent.

### Stroke definition

All included stroke events were symptomatic and were diagnosed using pre-established criteria, including clinical features and imaging evidence. First-ever (incident) stroke was defined as the first occurrence (based on medical records) of rapidly developing signs of a focal neurologic disturbance of presumed vascular etiology lasting >24 h [38].

Recurrent stroke was defined as a new stroke event occurring >28 days after the incident stroke. The stroke types included in this analysis were ischemic stroke (IS) and intracerebral hemorrhage (ICH). IS was defined as a thrombotic brain infarction, cardioembolic stroke, or lacunar infarct.

### Inclusion and exclusion criteria

Only patients who survived >28 days after the incident event were entered into the present analysis. Patients were included in the study if they participated in at least 5 years of follow-up, regardless of stroke recurrence. All patients with transient ischemic attacks, suspected stroke deaths without imaging evidence or confirmation by a neurologist, and silent strokes detected only by imaging were excluded from this study. Deaths were excluded if they occurred within 28 days of the incident stroke. Monthly follow-up evaluations were conducted; recurrent stroke events were classified using information from interviews (during follow-up visits or by telephone) with patients, next of kin, witnesses, and attending physicians.

### Event ascertainment and processes

The recruitment period was January 1, 1992 through December 31, 2012, with follow-up completed on December 31, 2017. During the surveillance period, all stroke events and all-cause deaths were registered and followed-up. Dates of death or emigration from the township were based on population registries. All changes in demographic information were recorded, including births, deaths, immigrations (due to marriages), and emigrations (due to entering a high school or university, or working in the city). Local residents who worked as seasonal workers in the city were included in the study as they regularly returned to the township for traditional festivals and during the farming season.

Stroke events were reported according to predefined procedures. First, local physicians reported initial stroke events to the community hospital within 24 h of onset. Second, community hospital physicians visited the surviving patients within 72 h to obtain clinical feature information and confirm the stroke. Each month, these physicians reported confirmed stroke events (diagnosed by imaging) to the quality management group in TMUGH; suspected events (no imaging performed) were also reported in a timely manner. Finally, as soon as possible, a neurologist identified suspected cases during door-to-door interviews.

The community hospital physicians and a neurologist collected information regarding stroke onset during interviews with the patient or patient’s family. The collected information included patient demographics, time of stroke onset, clinical signs, and previous stroke status. The neurologist also obtained other information, including therapy and post-discharge outcomes, during interviews with the survivors or their family members.

### Statistical methods

The study period was broken into 3stages:1992–1998, 1999–2005, and 2006–2012. Categorical variables are presented as numbers (%) and between-group differences were compared using chi-square tests (2 groups) or tendency chi-square tests (>2 groups). Continuous variables are presented as means (standard deviations), and between-group differences were compared using Student’s t-tests (2 groups). The incidence of recurrent strokes was calculated as the cumulative incidence of recurrence events within 5 years after the incident stroke. Trends in the age-standardized incidence of recurrent strokes are expressed as relative risks (RRs) and 95% confidence intervals (CIs). SPSS, version 19.0, for Windows (SPSS, Chicago, IL, USA) was used for the analyses; statistical significance was defined as P <0.05.

## References

[1] Coull AJ, Rothwell PM. Underestimation of the early risk of recurrent stroke: evidence of the need for a standard definition. Stroke. 2004; 35:1925–29. 10.1161/01.STR.0000133129.58126.67 15192248

[2] Wang Z, Li J, Wang C, Yao X, Zhao X, Wang Y, Li H, Liu G, Wang A, Wang Y. Gender differences in 1-year clinical characteristics and outcomes after stroke: results from the China National Stroke Registry. PLoS One. 2013; 8:e56459. 10.1371/journal.pone.0056459 23418571 PMC3572058

[3] Kissela BM, Khoury JC, Alwell K, Moomaw CJ, Woo D, Adeoye O, Flaherty ML, Khatri P, Ferioli S, De Los Rios La Rosa F, Broderick JP, Kleindorfer DO. Age at stroke: temporal trends in stroke incidence in a large, biracial population. Neurology. 2012; 79:1781–87. 10.1212/WNL.0b013e318270401d 23054237 PMC3475622

[4] Feigin VL, Forouzanfar MH, Krishnamurthi R, Mensah GA, Connor M, Bennett DA, Moran AE, Sacco RL, Anderson L, Truelsen T, O’Donnell M, Venketasubramanian N, Barker-Collo S, et al, and Global Burden of Diseases, Injuries, and Risk Factors Study 2010 (GBD 2010) and the GBD Stroke Experts Group. Global and regional burden of stroke during 1990-2010: findings from the Global Burden of Disease Study 2010. Lancet. 2014; 383:245–54. 10.1016/S0140-6736(13)61953-4 24449944 PMC4181600

[5] Lee S, Shafe AC, Cowie MR. UK stroke incidence, mortality and cardiovascular risk management 1999-2008: time-trend analysis from the General Practice Research Database. BMJ Open. 2011; 1:e000269. 10.1136/bmjopen-2011-000269 22021893 PMC3211058

[6] Redon J, Olsen MH, Cooper RS, Zurriaga O, Martinez-Beneito MA, Laurent S, Cifkova R, Coca A, Mancia G. Stroke mortality and trends from 1990 to 2006 in 39 countries from Europe and Central Asia: implications for control of high blood pressure. Eur Heart J. 2011; 32:1424–31. 10.1093/eurheartj/ehr045 21487117

[7] Vaartjes I, O’Flaherty M, Capewell S, Kappelle J, Bots M. Remarkable decline in ischemic stroke mortality is not matched by changes in incidence. Stroke. 2013; 44:591–97. 10.1161/STROKEAHA.112.677724 23212165

[8] Touzé E, Varenne O, Chatellier G, Peyrard S, Rothwell PM, Mas JL. Risk of myocardial infarction and vascular death after transient ischemic attack and ischemic stroke: a systematic review and meta-analysis. Stroke. 2005; 36:2748–55. 10.1161/01.STR.0000190118.02275.33 16254218

[9] Putaala J, Haapaniemi E, Metso AJ, Metso TM, Artto V, Kaste M, Tatlisumak T. Recurrent ischemic events in young adults after first-ever ischemic stroke. Ann Neurol. 2010; 68:661–71. 10.1002/ana.22091 21031581

[10] Giang KW, Björck L, Ståhl CH, Nielsen S, Sandström TZ, Jern C, Torén K, Rosengren A. Trends in risk of recurrence after the first ischemic stroke in adults younger than 55 years of age in Sweden. Int J Stroke. 2016; 11:52–61. 10.1177/1747493015607519 26763020

[11] Liu L, Wang D, Wong KS, Wang Y. Stroke and stroke care in China: huge burden, significant workload, and a national priority. Stroke. 2011; 42:3651–54. 10.1161/STROKEAHA.111.635755 22052510

[12] Zhao D, Liu J, Wang W, Zeng Z, Cheng J, Liu J, Sun J, Wu Z. Epidemiological transition of stroke in China: twenty-one-year observational study from the Sino-MONICA-Beijing Project. Stroke. 2008; 39:1668–74. 10.1161/STROKEAHA.107.502807 18309149

[13] Jiang B, Wang WZ, Chen H, Hong Z, Yang QD, Wu SP, Du XL, Bao QJ. Incidence and trends of stroke and its subtypes in China: results from three large cities. Stroke. 2006; 37:63–68. 10.1161/01.STR.0000194955.34820.78 16306469

[14] Wang J, Bai L, Shi M, Yang L, An Z, Li B, Zhao W, Gu H, Zhan C, Tu J, Ning X. Trends in age of first-ever stroke following increased incidence and life expectancy in a low-income Chinese population. Stroke. 2016; 47:929–35. 10.1161/STROKEAHA.115.012466 26869385

[15] Wang J, An Z, Li B, Yang L, Tu J, Gu H, Zhan C, Liu B, Su TC, Ning X. Increasing stroke incidence and prevalence of risk factors in a low-income Chinese population. Neurology. 2015; 84:374–81. 10.1212/WNL.0000000000001175 25540314

[16] Aarnio K, Haapaniemi E, Melkas S, Kaste M, Tatlisumak T, Putaala J. Long-term mortality after first-ever and recurrent stroke in young adults. Stroke. 2014; 45:2670–76. 10.1161/STROKEAHA.114.005648 25061076

[17] Pezzini A, Grassi M, Lodigiani C, Patella R, Gandolfo C, Zini A, Delodovici ML, Paciaroni M, Del Sette M, Toriello A, Musolino R, Calabrò RS, Bovi P, et al, and Italian Project on Stroke in Young Adults (IPSYS) Investigators. Predictors of long-term recurrent vascular events after ischemic stroke at young age: the Italian Project on Stroke in Young Adults. Circulation. 2014; 129:1668–76. 10.1161/CIRCULATIONAHA.113.005663 24508827

[18] Rutten-Jacobs LC, Maaijwee NA, Arntz RM, Schoonderwaldt HC, Dorresteijn LD, van der Vlugt MJ, van Dijk EJ, de Leeuw FE. Long-term risk of recurrent vascular events after young stroke: the FUTURE study. Ann Neurol. 2013; 74:592–601. 10.1002/ana.23953 23776015

[19] Boysen G, Truelsen T. Prevention of recurrent stroke. Neurol Sci. 2000; 21:67–72. 10.1007/s100720070098 10938183

[20] Anderson CS, Carter KN, Brownlee WJ, Hackett ML, Broad JB, Bonita R. Very long-term outcome after stroke in Auckland, New Zealand. Stroke. 2004; 35:1920–24. 10.1161/01.STR.0000133130.20322.9f 15178818

[21] Dhamoon MS, Sciacca RR, Rundek T, Sacco RL, Elkind MS. Recurrent stroke and cardiac risks after first ischemic stroke: the Northern Manhattan Study. Neurology. 2006; 66:641–46. 10.1212/01.wnl.0000201253.93811.f6 16534100

[22] Hardie K, Jamrozik K, Hankey GJ, Broadhurst RJ, Anderson C. Trends in five-year survival and risk of recurrent stroke after first-ever stroke in the Perth Community Stroke Study. Cerebrovasc Dis. 2005; 19:179–85. 10.1159/000083253 15644631

[23] Petty GW, Brown RD Jr, Whisnant JP, Sicks JD, O’Fallon WM, Wiebers DO. Survival and recurrence after first cerebral infarction: a population-based study in Rochester, Minnesota, 1975 through 1989. Neurology. 1998; 50:208–16. 10.1212/WNL.50.1.208 9443482

[24] Burn J, Dennis M, Bamford J, Sandercock P, Wade D, Warlow C. Long-term risk of recurrent stroke after a first-ever stroke. The Oxfordshire Community Stroke Project. Stroke. 1994; 25:333–37. 10.1161/01.STR.25.2.333 8303740

[25] Ning X, Sun J, Jiang R, Lu H, Bai L, Shi M, Tu J, Wu Y, Wang J, Zhang J. Increased stroke burdens among the low-income young and middle aged in rural China. Stroke. 2017; 48:77–83. 10.1161/STROKEAHA.116.014897 27924051

[26] National Bureau of Statistics of China. In China Statistical Yearbook—2016 (2016). Beijing: China Statistics Press; 2016.

[27] Tuttolomondo A, Pecoraro R, Simonetta I, Miceli S, Arnao V, Licata G, Pinto A. Neurological complications of Anderson-Fabry disease. Curr Pharm Des. 2013; 19:6014–30. 10.2174/13816128113199990387 23448452

[28] Tuttolomondo A, Pecoraro R, Simonetta I, Miceli S, Pinto A, Licata G. Anderson-Fabry disease: a multiorgan disease. Curr Pharm Des. 2013; 19:5974–96. 10.2174/13816128113199990352 23448451

[29] Wang J, Ning X, Yang L, Lu H, Tu J, Jin W, Zhang W, Su TC. Trends of hypertension prevalence, awareness, treatment and control in rural areas of northern China during 1991-2011. J Hum Hypertens. 2014; 28:25–31. 10.1038/jhh.2013.44 23739160

[30] Tuttolomondo A, Di Sciacca R, Di Raimondo D, Pedone C, La Placa S, Pinto A, Licata G. Effects of clinical and laboratory variables and of pretreatment with cardiovascular drugs in acute ischaemic stroke: a retrospective chart review from the GIFA study. Int J Cardiol. 2011; 151:318–22. 10.1016/j.ijcard.2010.06.005 20598761

[31] Di Raimondo D, Tuttolomondo A, Buttà C, Miceli S, Licata G, Pinto A. Effects of ACE-inhibitors and angiotensin receptor blockers on inflammation. Curr Pharm Des. 2012; 18:4385–413. 10.2174/138161212802481282 22283779

[32] Bergström L, Irewall AL, Söderström L, Ögren J, Laurell K, Mooe T. One-year incidence, time trends, and predictors of recurrent ischemic stroke in Sweden from 1998 to 2010: an observational study. Stroke. 2017; 48:2046–51. 10.1161/STROKEAHA.117.016815 28706114

[33] Lee M, Wu YL, Ovbiagele B. Trends in incident and recurrent rates of first-ever ischemic stroke in Taiwan between 2000 and 2011. J Stroke. 2016; 18:60–65. 10.5853/jos.2015.01326 26687123 PMC4747065

[34] Santalucia P, Baviera M, Cortesi L, Tettamanti M, Marzona I, Nobili A, Riva E, Fortino I, Bortolotti A, Merlino L, Roncaglioni MC. Epidemiologic trends in hospitalized ischemic stroke from2002 to 2010: results from a large Italian population-based study. J Stroke Cerebrovasc Dis. 2015; 24:1917–23. 10.1016/j.jstrokecerebrovasdis.2015.05.008 26051662

[35] Pennlert J, Eriksson M, Carlberg B, Wiklund PG. Long-term risk and predictors of recurrent stroke beyond the acute phase. Stroke. 2014; 45:1839–41. 10.1161/STROKEAHA.114.005060 24788972

[36] Lewsey J, Jhund PS, Gillies M, Chalmers JW, Redpath A, Briggs A, Walters M, Langhorne P, Capewell S, McMurray JJ, MacIntyre K. Temporal trends in hospitalisation for stroke recurrence following incident hospitalisation for stroke in Scotland. BMC Med. 2010; 8:23. 10.1186/1741-7015-8-23 20380701 PMC2859404

[37] Sjölander M, Eriksson M, Glader EL. Social stratification in the dissemination of statins after stroke in Sweden. Eur J Clin Pharmacol. 2013; 69:1173–80. 10.1007/s00228-012-1454-8 23187964

[38] Aho K, Harmsen P, Hatano S, Marquardsen J, Smirnov VE, Strasser T. Cerebrovascular disease in the community: results of a WHO collaborative study. Bull World Health Organ. 1980; 58:113–30. 6966542 PMC2395897

